# Associations between musical abilities and precursors of reading in preschool aged children

**DOI:** 10.3389/fpsyg.2015.01220

**Published:** 2015-08-17

**Authors:** Franziska Degé, Claudia Kubicek, Gudrun Schwarzer

**Affiliations:** Department of Developmental Psychology, Justus Liebig University Giessen, GiessenGermany

**Keywords:** musical abilities, precursors of reading, phonological awareness, working memory, preschoolers

## Abstract

The association between music and language, in particular, the overlap in their processing results in the possibility to use one domain for the enhancement of the other. Especially in the preschool years music may be a valuable tool to train language abilities (e.g., precursors of reading). Therefore, detailed knowledge about associations between musical abilities and precursors of reading can be of great use for designing future music intervention studies that target language-related abilities. Hence, the present study investigated the association between music perception as well as music production and precursors of reading. Thereby, not only phonological awareness, the mostly studied precursor of reading, was investigated, but also other precursors were examined. We assessed musical abilities (production and perception) and precursors of reading (phonological awareness, working memory, and rapid retrieval from long-term memory) in 55 preschoolers (27 boys). Fluid intelligence was measured and controlled in the analyses. Results showed that phonological awareness, working memory, and rapid retrieval from long-term memory were related to music perception as well as to music production. Our data suggest that *several* precursors of reading were associated with music perception as well as music production.

## Introduction

The non-musical benefits of music lessons have intrigued the public and fascinated researchers. However, the potential of music lessons to enhance cognitive abilities (e.g., IQ or memory) still remains under discussion. There are only few experiments that clearly demonstrated a causal relationship between music lessons and cognitive abilities (e.g., IQ; Schellenberg, 2004). The vast majority of studies only established an association between music lessons and cognitive abilities, but did not examine the effect of music lessons on cognitive abilities. However, regarding language-related abilities, several studies showed that music interventions could cause improvements. Particularly, intervention studies demonstrated that music training can enhance vocabulary (Moreno et al., 2011), reading (Moreno et al., 2009), and phonological awareness – a precursor of reading (Degé and Schwarzer, 2011). These results indicate that music interventions might be able to support the development of language-related abilities (e.g., reading and phonological awareness). Until now, the majority of studies have primarily focused on music perception abilities (i.e., rhythm, pitch, meter, and timbre) and their relationship to language-related abilities, mostly addressing only one precursor (i.e., phonological awareness). However, there are other precursors of reading that have been identified: working memory, and rapid retrieval from long-term memory (Jansen et al., 2002). But up to now there has not been much research concerning musical abilities and precursors of reading other than phonological awareness. Another issue concerns the inclusion of musical abilities: although musical abilities comprise perception as well as production abilities, the association between musical production abilities and precursors of reading is still understudied. Therefore, our exploratory study is aimed at investigating the association between musical perception abilities as well as musical production abilities and *several* precursors of reading, such as phonological awareness, working memory, and rapid retrieval from long-term memory.

### Explanations for the Associations between Music and Language

Explanations draw upon the functional overlap of brain structures that are involved in music and speech processing (Besson et al., 2011). It is assumed that domain-general abilities (abilities that are used for music as well as for speech processing) build the basis of the connection between music and language. Explanatory approaches only differ in the auditory features that they promote as the connecting domain-general abilities. Besson et al. (2011) assume that musicians have an enhanced sensitivity to auditory parameters (e.g., frequency and duration) that are important for music processing. Because these parameters are involved in music and speech processing, the higher sensitivity results in a more elaborated auditory perception of speech. This enhancement on lower levels of processing can also create an advantage for higher levels of speech processing (e.g., phonological processing).

While the former approach focuses on frequency and duration, another one postulates timing as the important acoustical feature. Tierney and Kraus (2014) put forward the precise auditory timing hypothesis that explains the connection between auditory motor entrainment and phonological skills. They assume that music training requires entrainment, and entrainment necessitates the precise perception of acoustic event timing. Hence, musical training over extended periods of time might result in higher timing precision in the automated representation of acoustic events in the auditory system. This higher precision also benefits speech sound perception, which is important for phonological skills. Taken together, it is highly likely that common auditory features trained by musical experience have a positive effect on speech processing. As different explanations focus on different auditory features, it might be possible that more mechanisms drive the effect of music training on language abilities (Tierney and Kraus, 2014).

### Music Training and Vocabulary

There is correlational (i.e., quasi-experimental) and experimental evidence of associations between music training and vocabulary. Piro and Ortiz (2009) found in a study with second-grade students that *music training* was associated with improvements in vocabulary. Also, musically trained 10-year-old children outperformed their untrained counterparts in vocabulary tasks (Forgeard et al., 2008). However, these correlational studies allowed no inferences about causation; it therefore remained unclear whether music training actually caused improvements in vocabulary. Nonetheless, there is one experimental study that used a computerized music-listening training as a potential intervention for vocabulary (Moreno et al., 2011). Preschoolers were pseudo-randomly assigned to music training or visual arts training. Before and after 4 weeks (20 days) of training, vocabulary was assessed. Only the music group showed increases from pre- to post-test on the vocabulary task, which unequivocally demonstrates that the music intervention indeed enhanced vocabulary.

### Music Aptitude, Music Training, and Reading

Regarding *music aptitude*, Anvari et al. (2002) showed that music perception abilities (pitch and rhythm) were related to reading abilities in 4-year-old children. This association remained reliable when phonological awareness was held constant. In 5-year-old children only pitch perception was associated with reading, whereas rhythm was not related to reading. The relationship between pitch perception and reading was again independent of phonological awareness. This independence might indicate that, apart from phonological awareness, other precursors of reading might mediate the association between musical abilities and reading. In 7- to 8-year-old children, Douglas and Willatts (1994) demonstrated an association between reading and pitch perception as well as rhythm perception.

Correlational research regarding *music training* and reading skills demonstrated an association between music training and spelling in 8- to 9-year-old children (Hille et al., 2011). Additionally, an association between music training and reading was revealed; this association disappeared when socioeconomic status (SES) was controlled (Hille et al., 2011). However, a study with 6- to 9-year-old children revealed an association between length of music training and reading comprehension even when SES was controlled (Corrigall and Trainor, 2011). Even some experimental or longitudinal studies supported the assumption of a causal relation between music training and readings skills. Douglas and Willatts (1994) performed a small scale intervention study. They trained the music group and a discussion control group for 6 months, and measured reading skills before and after the training. The music group showed small improvements in reading, whereas the control group did not improve from pre- to post-test. In a recent study with a larger sample size, Moreno et al. (2009) trained 8-year-old children either in music or painting and tested reading skills (i.e., reading of inconsistent words; inconsistent with respect to phoneme grapheme mapping and pronunciation) before and after 6 months of training. The music group improved its reading skills, while the painting group showed no improvements.

Two meta-analyses were conducted on music training and reading skills. Butzlaff (2000) reported a strong association between music training and reading with respect to correlational studies. Though, for experimental studies he found no reliable results because some studies demonstrated a positive effect of music training on reading skills, while others did not. However, a more recently conducted meta-analysis (Standley, 2008) did reveal a modest but significant positive effect of music training on reading skills.

### Music Aptitude, Music Training, and Phonological Awareness

Phonological awareness describes the insight into the phonological structure of language. It refers to the ability to analyze and manipulate language on two levels. On the word level, phonological awareness describes the ability to manipulate and analyze larger phonological units (e.g., rhyming and blending words). On the phoneme level, phonological ability refers to the ability to analyze and manipulate the individual sound units (phonemes) within a word. It has been shown that phonological awareness is an important precursor of later reading ability (Pratt and Brady, 1988; Bruck, 1992).

Phonological awareness is related to music aptitude as well as to music training. A few studies investigated the effect of *music training* on phonological awareness, and revealed that music training can indeed enhance phonological skills. In a quasi-experiment, Gromko (2005) investigated the effect of music training on phonological awareness. Children in the treatment kindergarten received music training for 4 months, while children in the control kindergarten received no treatment. Gromko (2005) revealed significantly greater gains in phonological awareness in the treatment kindergarten children than in the control kindergarten children. The pseudo-random assignment of the preschoolers to the treatment and the control group, however, precludes firm conclusions. Children were not assigned randomly on an individual basis, but the kindergartens were chosen to be the control or the treatment kindergarten. Therefore, children in the treatment group may have systematically differed (e.g., in SES) from children in the control group. Furthermore, the control group did not receive an alternative training. Degé and Schwarzer (2011) randomly assigned preschoolers to music production and perception training, phonological skills training, and sports training. Children in all three groups received training for 20 weeks. The phonological skills training as well as the music training enhanced phonological awareness, whereas the sports training did not. The advancement of phonological awareness in the music group and in the phonological skills group was mainly driven by improvements in phonological awareness on the word level. These results demonstrated unequivocally that music training could enhance phonological awareness.

Several studies revealed positive associations between *music aptitude* and phonological awareness. Huss et al. (2011) found a relationship between metrical perception and phonological awareness in a sample of 10-year-old dyslexic and non-dyslexic children. Also, Norton et al. (2005) found that audiation (the ability to hear, feel, and comprehend music for which the sound is not physically present) was correlated with phonological awareness. The test that was applied in this study comprises pitch perception and rhythm perception. Hence, phonological awareness was associated with a global music perception factor. Moreover, in a study with 4- and 5-year-old children, Lamb and Gregory (1993) investigated the association between phonological awareness and pitch as well as timbre perception. They found that pitch perception (but not timbre perception) was related to phonological awareness (Lamb and Gregory, 1993). The association between pitch perception and phonological awareness was reliable even when age and fluid intelligence was controlled. By testing the same age group as Lamb and Gregory (1993), Anvari et al. (2002) investigated the association between musical abilities and phonological awareness. They assessed melody perception, chord perception, chord analysis, rhythm perception, and rhythm production. However, they ran factor analyses and found that one factor for the 4-year-olds and two factors for the 5-year-olds represented the musical abilities best. Thus, for their further analyses, they created one music macro-variable that contained all assessed musical abilities for the 4-year-old children and two music variables (i.e., pitch and rhythm) for the 5-year-old children. For the 4-year-old children, the music macro-variable was correlated with phonological awareness, and for the 5-year-olds both music variables (i.e., pitch and rhythm) were correlated with phonological awareness. In sum, these data suggest that pitch perception as well as rhythm perception is associated with phonological awareness.

### Objectives

Music intervention studies showed that music training could enhance vocabulary, reading skills, and phonological awareness. An explanation could be that music interventions that mostly target music perception abilities enhanced music listening skills. This enhancement was then accompanied by improvements in speech perception and might have promoted some aspects of language processing (Corrigall et al., 2013). This language enhancing potential of music training might be particularly valuable. Therefore, analyzing the association between musical abilities and language-related abilities might be of great importance in order to understand relevant underlying mechanisms, and to design effective interventions for reading or phonological awareness. So far, studies investigating the association between musical abilities and language-related abilities focused on music perception abilities only. Thus, the first aim of our study was to examine the association between precursors of reading in preschoolers and music perception as well as music production. Furthermore, we applied a musical test battery that assessed music perception (melody perception, pitch perception, rhythm perception, meter perception, tone length perception) as well as music production (singing a song, rhythm production, meter production) on a detailed level. This detailed investigation represents a more complete picture of associations between musical abilities and precursors of reading. This will in turn help to identify reasonable tasks for music training programs that focus, for example, on the improvement of reading.

Anvari et al. (2002) revealed that there might be abilities other than phonological awareness involved in the association between musical abilities and reading skills. However, most studies have investigated only one precursor of reading (i.e., phonological awareness). Therefore, our second aim was to investigate the association between musical abilities and several precursors of reading (i.e., phonological awareness, working memory, rapid retrieval from long-term memory) in preschool children. This exploratory approach which comprises several correlational analyses will broaden our understanding of associations between musical abilities and precursors of reading.

## Materials and Methods

The study was conducted in full accordance with the Ethical Guidelines of the German Association of Psychologists (DGPs). In accordance with the ethical guidelines mentioned above informed consent was obtained from the parents for each participant.

### Participants

The sample comprised 55 preschoolers (27 boys, 28 girls; mean age = 75.13 months; SD = 4.02 months) from five different kindergartens in Giessen, Germany. Participants had a mean fluid intelligence score of *M* = 113.25 (SD = 11.06), see below. Hence, average fluid intelligence scores were higher than the published norms. The sample showed diversity with respect to parents’ education: for 40% of the children neither parent had a university degree, for 27.3% of the children one parent had a university degree, and for 32.7% of the children both parents had a university degree.

### Measures

Possible confounding variables such as age, gender, SES, and intelligence were assessed. As predictor variables musical abilities and as criterion variables precursors of reading were measured.

Parents completed a demographic questionnaire that asked for information about their education as one possible measure of SES. Mothers’ and fathers’ education was initially coded as a dichotomous variable (0 for “no university degree” and 1 for “a university degree”). For the statistical analyses, parents’ education was collapsed into a single variable: 0, 1, or 2 parents with a university degree. Although parental income or parental profession could also be used as a measure of SES, we decided to ask for parents’ education, because in former studies parents have been mostly willing to share this information. This questionnaire was also used to assess gender and age of the participants.

To measure intelligence, the culture fair test (CFT1; Weiß and Osterland, 1977), which measures fluid intelligence, was employed. The test consisted of five subtests (substitution, mazes, classification, similarities, and matrices) and was administered in groups that did not exceed six children. The duration of test administration was 60 min including instructions and breaks. Age norms were used to determine the intelligence score for each participant.

Precursors of reading were measured with the Bielefelder Screening (BISC; Jansen et al., 2002). The screening allows the assessment of different precursors of reading: phonological awareness, working memory, and rapid retrieval from long-term memory.

Phonological awareness was assessed with the following four subtests: rhymes, word segmentation, phoneme synthesis, and phoneme recognition. Two tests (rhymes and word segmentation) measured phonological awareness for large phonological units (words) and the other two subtests (phoneme synthesis and phoneme recognition) assessed phonological awareness for small phonological units (phonemes). Each subtest consisted of two to four practice items and 10 test items. In the rhymes task, children were asked whether two words rhyme or do not rhyme (e.g., Do train and rain rhyme?). Children were asked to segment words by clapping their hands in the word segmentation task. The phoneme synthesis task requested the synthesis of the initial sound and the remaining word (e.g., m-ouse) into one word. The phoneme recognition task required recognition of a particular phoneme in a word (e.g., Is there a “u” in elephant?). A composite score of all of the subtest scores was calculated. For the statistical analyses the subtest scores as well as the composite score were used.

Working memory was assessed with recall of non-sense words (e.g., gor-ki-ra-si-mi). In this task the children had to listen to a non-sense word and recall it immediately after listening. The test consisted of two practice items and 10 test items. Seven of these test items were four syllables long, two were five syllables long, and one test item was six syllables long. The practice items were three and four syllables long, respectively. If any syllable was recalled incorrectly or omitted the non-sense word was marked as incorrect. For each correctly recalled word children received one point.

Rapid retrieval from long-term memory was assessed with a speeded naming task. This task consisted of two parts. In the first part, children were asked to name the appropriate color of black and white fruits as fast as possible. Reaction time was measured and the amount of correct answers was registered. This task was designed to assess rapid retrieval from long-term memory. In the second part, children were asked to name the appropriate color of wrongly colored fruits (e.g., yellow salad or blue lemon). As in the first part, reaction time and correctness were registered. This task should assess interference of rapid retrieval from long-term memory. The interference score was built by subtracting the “correct answer and time score” from part one from the “correct answer and time score” from part two. This difference score (small difference indicating little interference) was then transformed into the interference score (high score indicating little interference).

Musical abilities (music perception and production abilities) were measured with the music screening for children (Jungbluth and Hafen, 2005). We applied five subtests to measure music perception: melody perception, pitch perception, rhythm perception, tone length perception, and meter perception. Each subtest consisted of 10 items with increasing difficulty. All subtests required same-different discriminations, wherein the position or direction of changes should be indicated. In the melody perception subtest, children were asked to identify a change and the position of the change in two consecutive melodies. On the pitch perception task, children had to decide whether the second tone was higher, lower, or the same as the first. In the subtest rhythm perception, children had to decide whether two short rhythmic patterns were the same or different and they had to indicate the position of the difference. In the subtest tone length perception, two tones of the same pitch were played to the children and they had to indicate whether the second tone was longer, shorter, or of same duration as the first. In the subtest meter perception, two different meters were presented. Each consisted of five beats. The children had to decide whether the second meter was faster, slower, or the same as the first. A music perception total score was built for each child by adding the scores reached in each subtest.

We used three subtests to assess music production abilities: singing a song, rhythm production, and meter execution. In the subtest singing a song, children learned and sang a 4-bar-song. Two independent raters analyzed the recorded performance. They rated melody contour, rhythm, starting tone, and intonation. The interrater reliability was *r* = 0.94. In the subtest rhythm production, 10 rhythms of increasing levels of difficulty were presented from a CD and reproduced by the children on a keyboard. This subtest was recorded and scored by two independent raters, as well. The interrater reliability was *r* = 0.96. On the meter execution subtest, children had to perform four different tasks, while they always listened to the same piece of music. Firstly, children had to walk in the meter of the musical piece. Secondly, children had to clap their hands in the meter of the musical piece. In task three and four, children had to clap their hands in the meter of the music and continue clapping in the correct meter when the music had stopped. They continued until they heard “stop” from the CD. All four tasks were recorded and were coded by two raters. The interrater reliability was *r* = 0.80. In addition to the subtest scores, a music production total score was built for each child by adding the scores reached in each subtest.

### Procedure

Prior to testing, the informed consent of the parents was attained. Additionally, the demographic questionnaire was sent to the participants and they were asked to hand them back to a person working in the kindergarten. This way the experimenter could collect them. All test sessions took place in the kindergarten during their daily routine. All kindergartens provided a quiet room for the test sessions. The intelligence test was performed in groups of five to six children with two experimenters present. One experimenter instructed the children, while the second experimenter made sure that the children remained seated and concentrated on their own sheet of paper. The precursors of reading were assessed in individual sessions. Two experimenters applied the tests that measured music perception abilities in groups of five to six children. Again one experimenter instructed the group, while the other experimenter made sure that the children were focused on the tasks. The items of the music perception test were presented via speakers and the children indicated their responses on a sheet of paper. Items were coded with little cartoons or pictures to guide the children through the test. In case the children had to indicate the positions of differences (melody and rhythm) they could mark on the sheet of paper the notes or drums, respectively. Music production abilities were assessed in individual test sessions. The assessments were performed on consecutive days. All in all, children participated in four group sessions (two sessions intelligence test, two sessions music perception tasks) and two individual sessions (one session precursors of reading, one session music production tasks). At the end of the project, each child received a present and a certificate for participation.

## Results

### Preliminary Analyses

We correlated possible confounding variables (age, gender, SES, and IQ) with music perception as well as production abilities. Only a significant correlation between IQ and music production (*r* = 0.321, *p* = 0.017) and IQ and music perception (*r* = 0.497, *p* = 0.000) was found. Age, gender, and SES were not significantly correlated with musical abilities (**Table 1**). Also possible confounding variables and precursors of reading were correlated. Age was significantly correlated with interference of rapid retrieval (*r* = 0.313, *p* = 0.020). SES was significantly correlated with working memory (*r* = 0.269, *p* = 0.047). IQ was significantly correlated with working memory (*r* = 0.325, *p* = 0.016). Gender did not show any significant correlations with any precursor of reading, for details see **Table 1**. Because IQ was significantly correlated with musical abilities and precursors of reading, it was controlled in further statistical analyses.

**Table 1:** Correlations among possible confounding variables [age, gender, socioeconomic status (SES), and IQ], musical abilities (music perception and music production), and precursors of reading (phonological awareness, working memory, and rapid retrieval from long-term memory).

	Age	Gender	SES	IQ
Music perception	0.137	0.119	0.091	0.497**
Music production	0.206	0.245	0.074	0.321*
Phonological awareness	0.086	0.057	-0.046	0.243
Working memory	0.120	-0.051	0.269*	0.325*
Rapid retrieval	0.155	0.069	-0.051	0.041
Interference of rapid retrieval	0.313	0.136	0.070	0.143

*p < 0.05, **p < 0.001.

### Principal Analyses

#### Correlations between Musical Abilities and Precursors of Reading

Correlations (with IQ partialed out) between musical abilities (music perception and production total scores) and precursors of reading revealed significant associations between musical abilities and phonological awareness, working memory, and rapid retrieval from long-term memory (**Table 2**).

**Table 2:** Correlations between precursors of reading and music production (total) and music perception (total).

	Phonological awareness	Working memory	Rapid retrieval	Interference of rapid retrieval
Music perception	**0.417 (0.002)**	**0.363 (0.007)**	0.156 (0.260)	**0.337 (0.013)**
Music production	**0.650 (0.000)**	**0.280 (0.040)**	0.019 (0.889)	0.262 (0.056)

*p-values in parentheses. Significant results in bold.*

Phonological awareness was correlated with music perception (*r* = 0.417, *p* = 0.002) and music production (*r* = 0.650, *p* = 0.000). Also, working memory was correlated significantly with music perception (*r* = 0.363, *p* = 0.007) as well as music production abilities (*r* = 0.280, *p* = 0.040). We observed a significant correlation for interference of rapid retrieval and music perception (*r* = 0.337, *p* = 0.013). Higher scores in music perception (total) were associated with less interference in rapid retrieval. Music production was not significantly correlated with interference of rapid retrieval (*r* = 0.262, *p* = 0.056). In further analyses the associations between musical abilities and precursors of reading were explored in more detail.

#### Correlations between Music Perception, Music Production, and Phonological Awareness

The more detailed (on subtest level) analyses showed that phonological awareness total score was significantly correlated (IQ controlled) with pitch perception (*r* = 0.321, *p* = 0.018), rhythm perception (*r* = 0.335, *p* = 0.018), and tone length perception (*r* = 0.322, *p* = 0.018). The subtests of phonological awareness on the word level were significantly correlated with rhythm perception (rhymes: *r* = 0.320, *p* = 0.018) and marginally significantly correlated with tone length perception (rhymes: *r* = 0.264, *p* = 0.053) and pitch perception (word segmentation: *r* = 0.269, *p* = 0.050). For the subtests regarding phonological awareness on the phoneme level only a correlation between phoneme recognition and tone length perception (*r* = 0.347, *p* = 0.010) was revealed. All the other correlations between phonological awareness and music perception were not significant (see **Table 3** for more details). The phonological awareness total score was significantly correlated with singing a song (*r* = 0.529, *p* = 0.000) and rhythm production (*r* = 0.632, *p* = 0.000). Both phonological awareness subtests on the word level were significantly correlated with singing a song (rhymes: *r* = 0.347, *p* = 0.010; word segmentation: *r* = 0.344, *p* = 0.011) and rhythm production (rhymes: *r* = 0.332, *p* = 0.014; word segmentation: *r* = 0.473, *p* = 0.000). Additionally, rhymes were also significantly correlated with meter execution (*r* = 0.443, *p* = 0.001). Only one subtest operating on the phoneme level was correlated with singing a song. Phoneme synthesis was significantly correlated with singing a song (*r* = 0.387, *p* = 0.004), whereas the correlation between phoneme recognition and singing a song was not significant (*r* = 0.257, *p* = 0.061). However, phoneme recognition was significantly correlated with rhythm production (*r* = 0.391, *p* = 0.003). None of the other correlations between phonological awareness and music production reached significance (see **Table 3**).

**Table 3:** Associations between phonological awareness (total score, rhymes, word segmentation, phoneme synthesis, phoneme recognition) and the subtests of music perception as well as the subtests of music production.

	Music perception					Music production		
	Melody perception	Pitch perception	Rhythm perception	Tone length perception	Meter perception	Singing a song	Rhythm production	Meter execution
Phonological awareness total	0.093 (0.505)	**0.321** **(0.018)**	**0.335** **(0.013)**	**0.322** **(0.018)**	0.246 (0.073)	**0.529 (0.000)**	**0.632** **(0.000)**	0.205 (0.138)
Rhymes	0.013 (0.928)	0.189 (0.172)	**0.320** **(0.018)**	**0.264** **(0.053)**	0.125 (0.369)	**0.347 (0.010)**	**0.332** **(0.014)**	**0.443 (0.001)**
Word segmentation	0.010 (0.945)	**0.269** **(0.050)**	0.170 (0.219)	0.110 (0.427)	0.154 (0.265)	**0.344 (0.011)**	**0.473** **(0.000)**	-0.014 (0.922)
Phoneme synthesis	0.138 (0.319)	0.104 (0.453)	0.108 (0.437)	0.139 (0.317)	0.214 (0.120)	**0.387 (0.004)**	0.227 (0.098)	-0.054 (0.700)
Phoneme recognition	0.164 (0.237)	0.114 (0.410)	0.231 (0.093)	**0.347** **(0.010)**	0.166 (0.230)	0.257 (0.061)	**0.391** **(0.003)**	0.175 (0.206)

*p-values in parentheses. Significant results in bold.*

#### Correlations between Music Perception, Music Production, and Working Memory

We calculated partial correlations with IQ controlled between working memory and the music perception subtests. Working memory was associated significantly with rhythm perception (*r* = 0.435, *p* = 0.001). For the other subtests (melody perception, pitch perception, tone length perception, and meter perception) no significant correlations were found (**Table 4**). Partial correlations between the music production subtests (singing a song, rhythm production, and meter execution) and working memory revealed a marginal significant relationship between rhythm production and working memory (*r* = 0.265, *p* = 0.053). Singing a song and meter execution were not significantly correlated with working memory (**Table 4**).

**Table 4:** Associations among working memory and the subtests of music perception as well as the subtests of music production.

	Music perception					Music production		
	Melody perception	Pitch perception	Rhythm perception	Tone length perception	Meter perception	Singing a song	Rhythm production	Meter execution
Working memory	0.147 (0.290)	0.102 (0.456)	**0.435** **(0.001)**	0.225 (0.102)	0.214 (0.120)	0.208 (0.131)	**0.265** **(0.053)**	0.154 (0.267)

*p-values in parentheses. Significant results in bold.*

#### Correlations between Music Perception, Music Production, and Interference of Rapid Retrieval from Long-Term Memory

Partial correlations (IQ controlled) between interference of rapid retrieval from long-term memory and music perception revealed a significant association with rhythm perception (*r* = 0.344, *p* = 0.011). No significant associations between interference of rapid retrieval from long-term memory and any other tested music perception ability were found (see **Table 5** for details). With respect to music production abilities, only rhythm production was significantly correlated with interference of rapid retrieval from long-term memory (*r* = 0.295, *p* = 0.030). Neither singing a song nor meter execution was significantly related with interference of rapid retrieval from long-term memory (see **Table 5**).

**Table 5:** Associations among interference of rapid retrieval from long-term memory and the subtests of music perception as well as the subtests of music production.

	Music perception					Music production		
	Melody perception	Pitch perception	Rhythm perception	Tone length perception	Meter perception	Singing a song	Rhythm production	Meter execution
Interference of rapid retrieval from long-term memory	0.101 (0.465)	0.092 (0.510)	**0.344** **(0.011)**	0.191 (0.167)	0.228 (0.098)	0.158 (0.253)	**0.295** **(0.030)**	0.196 (0.157)

*p-values in parentheses. Significant results in bold.*

Although this study was exploratory in nature and therefore several correlations were calculated, it should be taken into account that this exploratory approach affects the alpha level. Because in the principal analyses 64 correlations were calculated, it might be reasonable to adjust the alpha level. On an adjusted alpha level (*p* = 0.0008) only four correlations remained significant: the correlation between music production and phonological awareness total score, the correlation between singing a song and phonological awareness total score, the correlation between rhythm production and phonological awareness total score, and the correlation between rhythm production and word segmentation.

## Discussion

In the present study, we investigated associations between music perception as well as music production and precursors of reading. In particular, we assessed musical abilities on a detailed (subtest) level and examined their associations with several precursors of reading (phonological awareness, working memory, and rapid retrieval from long-term memory).

The total scores of music perception as well as of music production were associated with phonological awareness. Furthermore, we found correlations between the music perception total score and working memory as well as between the music production total score and working memory. Finally, the music perception total score correlated significantly with interference of rapid retrieval from long-term memory.

All in all, our results indicated that music production as well as music perception was associated with several precursors of reading. Thus, our study complements already existing studies by showing associations with music production abilities. With respect to production abilities, rhythm production was associated with three of the precursors, singing a song was correlated with all phonological awareness subtests, and meter execution showed only one significant relationship with rhymes, one subtest of phonological awareness. Our assessment of several precursors of reading and their relationship to musical abilities showed that above and beyond phonological awareness, working memory, and interference of rapid retrieval from long-term memory were associated with musical abilities. Taken together, our data suggest that there are several links between musical abilities and precursors of reading.

In a next step we explored the revealed associations between musical abilities and phonological awareness, working memory, and interference of rapid retrieval from long-term memory in more detail (i.e., on the subtest level).

Phonological awareness on the word level (rhymes and word segmentation) was correlated with pitch perception (marginally), rhythm perception, and tone length perception (marginally). Regarding phonological awareness on the word level and music production abilities, we found associations between singing a song, rhythm production, and meter execution. Phonological awareness on the phoneme level (phoneme recognition) was correlated with tone length perception. Furthermore, we revealed a significant correlation between phonological awareness on the phoneme level (phoneme recognition) and rhythm production and a significant correlation between phonological awareness on the phoneme level (phoneme synthesis) and singing a song. Thus, first of all, our study is in accordance with earlier findings that indicate a relationship between phonological awareness and musical abilities (Lamb and Gregory, 1993; Anvari et al., 2002). Furthermore, our results demonstrated that phonological awareness on the word level is involved in more associations with musical abilities than phonological awareness on the phoneme level. This fits to the results by Degé and Schwarzer (2011), who found an improvement especially for phonological awareness on the word level after a music training program. Our research indicates that more associations between phonological awareness on the word level and musical abilities are evident. Thus, it might have been easier to observe an effect of music training on phonological awareness on the word level in the Degé and Schwarzer study. Hence, musical abilities had more ways of interacting with phonological awareness on the word level. Therefore, a positive effect might have emerged earlier for phonological awareness on the word level than for phonological awareness on the phoneme level. Additionally, it is also possible that music training is not suitable to train phonological awareness on the phoneme level. However, we also found associations between phonological awareness on the phoneme level and musical abilities. Therefore, it should be possible to train phonological awareness on the phoneme level with a music training program. Because we found only few associations between musical abilities and phonological awareness on the phoneme level, it could be speculated that these associations might be weaker as compared to the word level, which in turn suggests that simply increasing training length might drive effects of music training on phonological awareness on the phoneme level. It remains to future research to test this specific hypothesis. Like Lamb and Gregory (1993), our results showed an association between phonological awareness and pitch perception that remained reliable after controlling for fluid intelligence. Hence, this association was not due to the influence of a third variable (i.e., fluid intelligence), but a direct association between pitch perception and phonological awareness. Contradictory to the results by Lamb and Gregory (1993), we found also reliable associations between phonological awareness and rhythm perception as well as rhythm production. This finding, though, is supported by the results of Anvari et al. (2002), who also found associations between pitch as well as rhythm and phonological awareness. Taken together, pitch and rhythm seem to be related to phonological awareness. Thus, both aspects of musical abilities might contribute to positive effects of music training on phonological awareness.

With respect to meter, we only found a significant relationship between meter production and phonological awareness. Meter perception was not significantly correlated with phonological awareness. Hence, our results are only partly in accordance with the study by Huss et al. (2011); in so far that both studies found a link between meter and phonological awareness. Huss et al. (2011) found correlations between phonological awareness and metrical perception. Interestingly, we revealed an association between phonological awareness and meter production (i.e., meter execution). Possibly, the difference in the applied meter perception tasks was responsible for the slightly different results. In the study by Huss et al. (2011) beats per minutes remained stable and only the accents were changed between consecutive stimuli, whereas in the task we applied two sequences differed in beats per minutes and not in accents. In the light of the precise auditory timing hypothesis (Tierney and Kraus, 2014) it is surprising that meter did not show more associations with several aspects of phonological awareness. Because meter execution and meter perception heavily rely on a precise perception of auditory timing, someone might have expected that meter is strongly related to phonological awareness.

For working memory results regarding perception and production showed an association between working memory and the rhythm subtests. Working memory was correlated with rhythm perception and rhythm production (marginally significant). These findings are in line with the results of Anvari et al. (2002). They showed an association between musical abilities and working memory. Considering the applied tasks to assess rhythm perception (compare two drum sequences), rhythm production (reproducing a rhythm on a keyboard), and working memory (reproduce non-sense words), it seems reasonable to conclude that all of them were processed by the phonological loop (i.e., the subsystem responsible for auditory/verbal input of Baddeley’s working memory model; Baddeley, 1986). Therefore, the phonological loop was possibly the common basis of these tasks and reflected in task outcome. This might indicate that in intervention studies music training might have trained phonological loop processes and thereby produced a benefit for language processing. Moreover, the rhythm perception and production tasks were to some extent quite similar. For both of them the children had to keep in mind a rhythm and either reproduce it or compare it to a second one. Thus, it comes as no surprise that rhythm perception and rhythm production tasks are related to the same precursor of reading; working memory. However, it might not be possible to generalize our findings to all kinds of working memory tasks. Because the task we applied uses non-sense words to assess working memory, it relies not only on working memory capacity but also on phonological processing and articulatory acuity. For example, if a child is not able to reproduce all letters of a syllable correctly this might be due to working memory capacity, but it is also possible that this child has difficulties with phonological processing. In the context of working memory as a precursor of reading it might be reasonable to use language material (non-sense words) in the working memory task. However, to claim a general link between rhythmical abilities and working memory future studies should investigate this association by using tests that do not rely on phonological abilities.

Interference of rapid retrieval from long-term memory was correlated with rhythm perception as well as with rhythm production. Hence, for interference of rapid retrieval from long-term memory associations with perception and production showed the same pattern of results. To the best of our knowledge our study is the first study that demonstrated an association between musical abilities and interference of rapid retrieval from long-term memory. As mentioned above, the rhythm tasks placed similar cognitive demands on the children. Therefore, it is plausible that they showed comparable relationships. This result again points toward the importance of memory for language processing. Although the task assessed rapid retrieval from long-term memory, it might be speculated that again a subsystem of working memory (the central executive) could build the common ground of the music and the language task. The central executive is, among other things, responsible for providing a link between working memory and long-term memory (Baddeley, 2007). Furthermore, the nature of the task (speeded naming of color incongruent fruits) is comparable to other set shifting tasks that typically assess central executive. Hence, the central executive, in particular set shifting abilities, might be important for musical abilities and rapid retrieval from long-term memory. Indeed, there is correlational evidence of an association between set shifting and music lessons (Degé, et al., 2011).

With regard to the design of music interventions studies focusing on enhancing precursors of reading, our results point out that music training should target music listening skills as well as music production skills. A combination of perception and production tasks will probably provide a more successful training of precursors of reading. Our most important finding is that music production should be part of a music training program. Additionally, our results show that rhythm perception and production tasks may be a powerful tool to enhance memory-related precursors of reading. Moreover, the present results suggest that not only pitch perception in the sense of discriminating pitches of different frequencies, but also discriminating different length of pitches may be important to train in a music intervention. Lastly, body movements, as required in meter execution, may be helpful in training phonological awareness. However, they probably play only a minor role, because meter execution was only related to the rhymes subtest of phonological awareness. It is important to note that all of the above mentioned suggestions were inspired by correlational data. Our study provides no evidence of a causal effect of music training on the mentioned precursors of reading, but sheds light on the path a music training might take to improve precursors of reading.

As already mentioned, our study aimed at analyzing associations between musical abilities and precursors of reading on a detailed level. Therefore, a detailed and exploratory approach was chosen. However, there is a tradeoff between the detailed picture we could show and the high amount of correlations tested. Due to several comparisons the level of significance should be adjusted. If the alpha level is adjusted, only associations between music production and phonological awareness remain reliable. Although in our analyses alpha inflation is a problem, we believe that this approach is seminal. It draws a more complete picture of association between musical abilities and precursors of reading than former research has done. Moreover, for future studies it is now possible to test specific relations and hypothesis.

Future studies should replicate our results with a larger sample size and should extend them by using different measures to assess musical abilities as well as precursors of reading to investigate the stability of the revealed associations. Finally, it remains to future research to employ an experimental design to allow inferences about causation.

## Conflict of Interest Statement

The authors declare that the research was conducted in the absence of any commercial or financial relationships that could be construed as a potential conflict of interest.
